# Selective and reversible suppression of intestinal stem cell differentiation by pharmacological inhibition of BET bromodomains

**DOI:** 10.1038/srep20390

**Published:** 2016-02-09

**Authors:** Akifumi Nakagawa, Curtis E. Adams, Yinshi Huang, Sulaiman R. Hamarneh, Wei Liu, Kate N. Von Alt, Mari Mino-Kenudson, Richard A. Hodin, Keith D. Lillemoe, Carlos Fernández-del Castillo, Andrew L. Warshaw, Andrew S. Liss

**Affiliations:** 1 Department of Surgery and The Andrew L. Warshaw Institute for Pancreatic Cancer Research, Massachusetts General Hospital, Boston MA, USA; 2 Department of Pathology, Massachusetts General Hospital, Boston MA, USA

## Abstract

Absorptive and secretory cells of the small intestine are derived from a single population of Lgr5-expressing stem cells. While key genetic pathways required for differentiation into specific lineages have been defined, epigenetic programs contributing to this process remain poorly characterized. Members of the BET family of chromatin adaptors contain tandem bromodomains that mediate binding to acetylated lysines on target proteins to regulate gene expression. In this study, we demonstrate that mice treated with a small molecule inhibitor of BET bromodomains, CPI203, exhibit greater than 90% decrease in tuft and enteroendocrine cells in both crypts and villi of the small intestine, with no changes observed in goblet or Paneth cells. BET bromodomain inhibition did not alter the abundance of Lgr5-expressing stem cells in crypts, but rather exerted its effects on intermediate progenitors, in part through regulation of Ngn3 expression. When BET bromodomain inhibition was combined with the chemotherapeutic gemcitabine, pervasive apoptosis was observed in intestinal crypts, revealing an important role for BET bromodomain activity in intestinal homeostasis. Pharmacological targeting of BET bromodomains defines a novel pathway required for tuft and enteroendocrine differentiation and provides an important tool to further dissect the progression from stem cell to terminally differentiated secretory cell.

The small intestine is comprised of a heterogeneous population of cells that can be classified into two broad groups, absorptive and secretory cells^1^,^2^. Absorptive cells primarily function to absorb nutrients and consist of a single cell type, enterocytes, which comprise 90% of the intestinal epithelium. The secretory group contains four cell types: goblet, Paneth, enteroendocrine, and tuft cells. Goblet cells are the most numerous of these cells and secrete mucins to protect the intestinal epithelium from harmful contents of the lumen^3^. Paneth cells function, in part, by secreting antimicrobials into the lumen^4^,^5^. Unlike other secretory cells, Paneth cells are restricted to the intestinal crypts, where they serve a key role in the stem cell niche^4^. Enteroendocrine cells secrete hormones that regulate the digestive process and are found sparsely throughout the intestinal epithelium^6^. Tuft cells are also found in small numbers in the intestinal epithelium. Although the exact function of these cells remains unclear, they appear to serve as chemosensory cells^7^.

Long-lived, multipotent Lgr5 + stem cells are located at the base of intestinal crypts and are the source of all intestinal cell types^8^. They give rise to transient amplifying (TA) cells, whose distinct genetic programs establish the ultimate cell type produced. Expression of Atoh1 in TA cells determines goblet, enteroendocrine, and Paneth cell type fate, while Hes1 expression inhibits Atoh1 and results in cells destined for enterocyte differentiation^9^. Downstream programs that determine cell fate within the secretory lineage have also been described. Goblet and Paneth cell differentiation depends on expression of the transcription factor Spdef^10^. Ngn3 is uniquely expressed in the TA cells responsible for enteroendocrine cell differentiation, and Ngn3 activity is required for this event^11^. The mechanisms regulating tuft cell differentiation are poorly defined; however, unlike other secretory cells, the fate of tuft cells is independent of Atoh1 signaling, and they are derived from Gfi1b expressing progenitor cells^1^,^12^.

While the genetic programs determining intestinal cell fate have been elucidated in recent years, little progress has been made in understanding the epigenetic regulation required for transition from multipotent stem cells to the various terminally differentiated cell types. Members of the BET family of chromatin adaptors are key epigenetic regulators in many tissues, and a variety of small molecule inhibitors of BET bromodomains are in clinical trials for cancer treatment^13^. BET proteins (Brd2, Brd3, Brd4, and BrdT) contain tandem bromodomains that allow for binding to acetylated lysines on target proteins to regulate gene expression^14^. A recent report demonstrates that genetic suppression of Brd4 expression disrupts tissue homeostasis in multiple organs in adult mice, most notably inducing stem cell loss in the small intestine^15^. In this study, we interrogate the role of BET proteins in intestinal stem cell differentiation using pharmacological inhibition of BET bromodomains.

## Results

### BET proteins are predominantly expressed in the crypts of the small intestine

**Figure 1:**
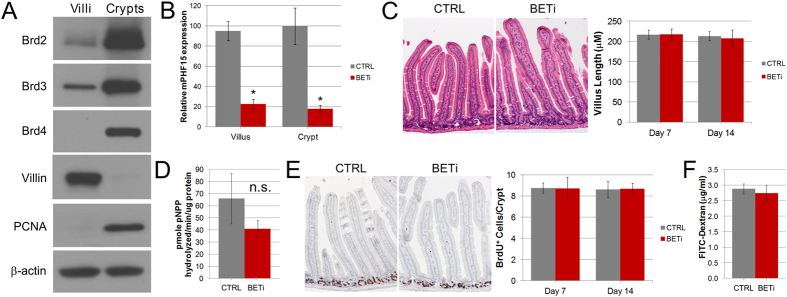
BET bromodomain inhibition does not alter the gross structure of the small intestine. (**A**) Western blot analysis of Brd2, Brd3, and Brd4 in whole cell lysates from preparations of crypts and villi from the jejunum. The predominant expression of Villin and PCNA in the villi and crypts, respectively, served as controls for the proper enrichment of these compartments. β-actin served as a loading control. Results are representative of three independent experiments. (**B**) RNA was isolated from purified villi and crypts from the jejunum of mice treated with vehicle control or BETi for 24 hours and the expression of PHF15 was evaluated. The average (±std. dev.) relative expression from three mice is shown. Differences in the expression that were statistically significant (p < 0.05) are indicated by an asterisk. (**C**) H&E images of the small intestine of mice after 14 days of treatment with BETi or vehicle control. The villus height (avg. ± std. dev.) measured from 3 control and BETi treated mice are shown. (**D**) Intestinal alkaline phosphatase activity was measured using p-nitrophenyl phosphate (pNPP) as a substrate with cell lysates from the small intestine of mice harvested after 14 days of treatment with BETi or vehicle control. The averages (±std. dev.) from three mice are shown. The differences in pNPP activity were not significant (n.s., p = 0.12). (**E**) Representative images of immunohistochemistry for BrdU incorporation in the crypts of the small intestine in mice after 7 and 14 days of treatment with BETi or vehicle control. The average (±std. dev.) number of BrdU positive cells per crypt is shown. (**F**) Intestinal permeability was determined serum levels of FITC-dextran after gastric gavage in mice after 14 days of treatment with vehicle control or BETi. The averages (±std. dev.) from 3 mice are shown.

To investigate how BET family members contribute to the biology of the murine small intestine, we first examined their relative expression and distribution. Enriched populations of crypts and villi were isolated from the jejunum of mice and whole cell lysates employed in Western blot analysis. These studies demonstrated that Brd2 and Brd3 were highly expressed in crypts and to lesser extent in the villi (Fig. 1A). In contrast, Brd4 was exclusively expressed in the crypts of the small intestine.

To determine the functional importance of BET family members to the small intestine, we employed the BET bromodomain inhibitor CPI203 (BETi). To verify inhibition of BET bromodomains within the small intestine, mice were administered three doses (10 mg/kg) of BETi in 24 hours. Four hours after the last dose, villi and crypts of the small intestine were isolated and qPCR performed to examine the expression of PHF15, a gene that is downregulated upon BET bromodomain inhibition in a wide variety of cell types^16^,^17^,^18^. PHF15 expression was decreased by more than 75% in both villi and crypts of mice treated with BETi relative to control, demonstrating effective delivery of BETi throughout the small intestine (Fig. 1B). To examine the effects of sustained BET bromodomain inhibition on the small intestine, mice were administered BETi or vehicle control twice daily for 7 or 14 days. Histological examination of the small intestine did not identify gross differences between control and BETi treated mice (Fig. 1C). Additionally, BET bromodomain inhibition did not significantly alter IAP activity (p = 0.12), which is consistent with proper differentiation of the enterocyte population of the small intestine (Fig. 1D). BrdU incorporation experiments demonstrated equivalent proliferative capacity in crypts between control and BETi treated mice, suggesting that BET bromodomain inhibition does not grossly affect crypt function (Fig. 1E). Consistent with the lack of obvious changes to the intestinal crypts and villi, intestinal permeability was not altered after 14 days of BETi treatment (Fig. 1F). Taken together, these results suggest that BET bromodomain inhibition does not affect the general structure of the small intestine.

### Administration of BETi selectively suppresses intestinal stem cell differentiation

**Figure 2:**
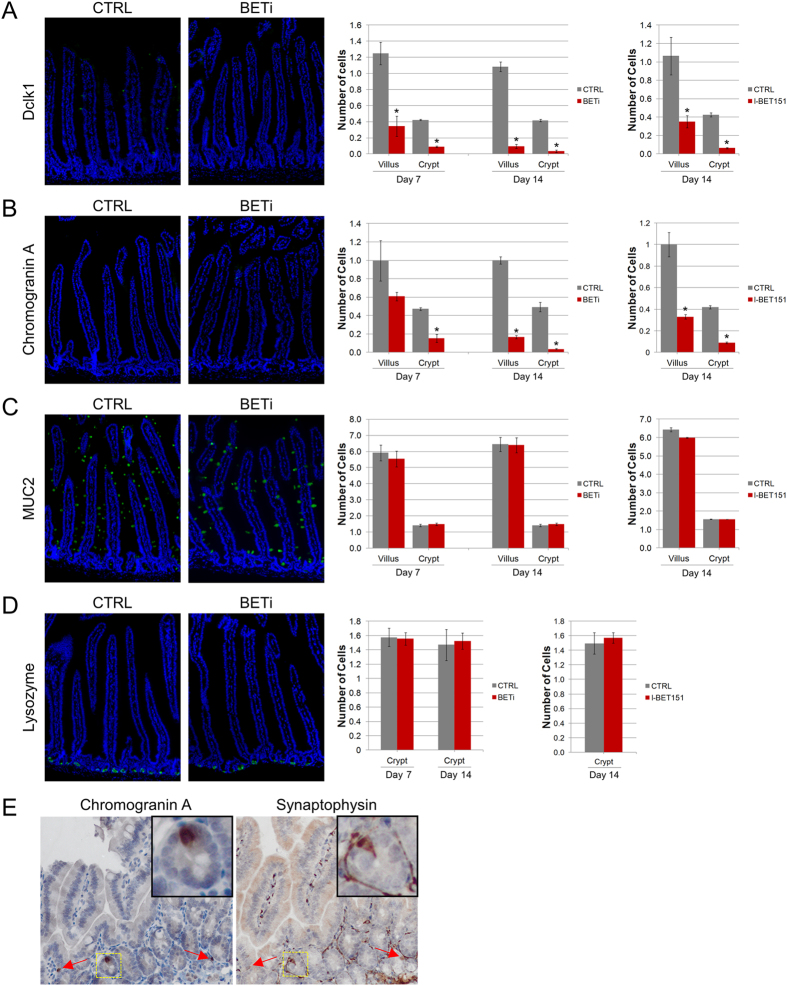
BET bromodomain inhibition results in the loss of tuft and enteroendocrine cells. Mice were administered vehicle control or BETi (10 mg/kg) twice daily for 7 or 14 days. (**A–D**) Representative sections of the jejunum were evaluated by immunofluorescence for the presence of tuft (Dclk1), enteroendocrine (chromogranin A), goblet (Muc2), and Paneth (lysozyme) cells. The average (±std. dev.) of each cell type observed in 100 villi and crypts in each of three mice from control and BETi treated groups are shown on the right. Statistically significant differences in cell counts are indicated by an asterisk (p ≤ 0.001). Mice were administered vehicle control or I-BET151 (30 mg/kg) once daily for 14 days. Representative sections of the jejunum were evaluated by immunohistochemistry for the Dclk1), chromogranin A, Muc2, and lysozyme. The average (±std. dev.) of each cell type observed in at least 200 villi and crypts from two control and three I-BET151 treated groups are shown in the graphs on the right. Statistically significant differences in cell counts are indicated by an asterisk (p ≤ 0.001). (**E**) Representative serial sections of the jejunum were evaluated by immunohistochemistry for enteroendocrine markers chromogranin A (left) and synaptophysin (right). Positively stained cells colocalizing between sections are indicated by arrows. The area within the dashed yellow box is shown in the inset of each photograph.

Since the expression of BET proteins was highly enriched in the crypts, we hypothesized that these proteins may contribute to the function of progenitor cells found in this compartment. Therefore, we examined whether BET bromodomain inhibition resulted in changes in the abundance of the secretory cell types of the intestinal epithelium. Administration of BETi for 7 days resulted in a dramatic decrease in the number of Dclk1 + tuft cells in both villi and crypts of the small intestine (Fig. 2A; 72% and 79% decrease; p = 0.001 and p < 0.0001, respectively). When BETi treatment was extended to 14 days, greater than 90% reduction in tuft cell numbers was seen in both villi and crypts. As active-caspase 3 staining did not identify apoptotic cells in the villi of BETi treated mice, the decrease in tuft cell number is likely due to a block in tuft cell formation. Supporting this idea, the kinetics of the loss of tuft cells after BETi treatment are consistent with a previous report that demonstrate tuft cells require up to 14 days to migrate from the crypt through the villus^19^.

Enteroendocrine cell numbers also decreased after BET bromodomain inhibition; although with slower kinetics than observed for tuft cells (Fig. 2B). After 7 days of BETi treatment, a 68% decrease in chromogranin A-expressing enteroendocrine cells was observed in the crypts (p < 0.001), while the small decrease observed in the villi was not statistically significant (p = 0.08). After 14 days of BET bromodomain inhibition, the number of enteroendocrine cells in both villi and crypts were significantly (p < 0.001) reduced to levels comparable with tuft cells at the same time point (83% and 93%, respectively). The time between the loss of enteroendocrine cells in the crypt and their corresponding loss in the villi is consistent with the lifespan of these cells, suggesting a block in enteroendocrine cell differentiation in the crypt^20^. Consistent with this, we observed a similar staining pattern with a second marker for enteroendocrine cells, synaptophysin, indicating that the reduction in chromogranin A positive cells reflects a decreased abundance of enteroendocrine cells, as opposed to their altered composition in response to BET bromodomain inhibition (Fig. 2E).

In contrast to the dramatic reduction in tuft and enteroendocrine cells, no changes in the number of goblet or Paneth cells were observed after 7 or 14 days of BET bromodomain inhibition (Fig. 2C,D). Furthermore, no changes in these cell numbers are seen even after 28 days of BETi treatment (data not shown). The selective alterations in the composition of intestinal secretory cells were not unique to the small molecule inhibitor CPI203. Mice treated for 14 days with a structurally unrelated BET bromodomain inhibitor, I-BET151, also exhibited reductions in tuft (67% villi and 85% in crypts) and enteroendocrine (67% in villi and 79% in crypts) cells in the small intestine (Fig. 2A,B, right panels)^21^. I-BET151 treatment did not alter the goblet or Paneth cell populations (Fig. 2C,D, right panels). Taken together, these results suggest that BET bromodomains are selectively important in tuft and enteroendocrine cell differentiation.

### The effects of BET bromodomain inhibition are reversible

**Figure 3:**
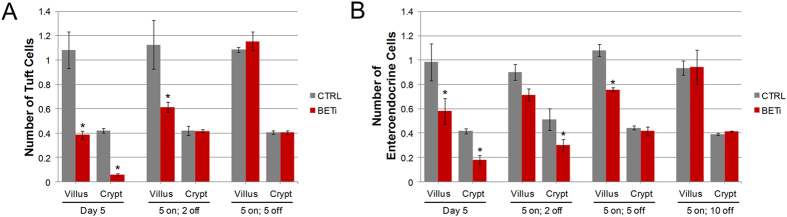
Pharmacological inhibition of tuft and enteroendocrine cell formation is reversible. Mice were administered vehicle control or BETi (10 mg/kg) twice daily for 5 days, and allowed to recover for 2, 5, or 10 days. The average (±std. dev.) of number of tuft cells (**A**) and enteroendocrine cells (**B**) observed in 100 villi and crypts in each of three mice from control and BETi treated groups are shown. Statistically significant differences in cell counts are indicated by an asterisk (p ≤ 0.02).

Tuft and enteroendocrine cells play key physiological and homeostatic roles in the small intestine; long term depletion of these cells is likely to have detrimental effects. Therefore, we investigated whether cessation of BET bromodomain inhibition allows for the restoration of the tuft and enteroendocrine cell populations. Mice were treated with vehicle control or BETi for 5 days followed by 2, 5, or 10 days of recovery (Fig. 3). Five days of BET bromodomain inhibition resulted in similar reductions in the tuft and enteroendocrine cell populations as seen at 7 days (p < 0.002). After 2 days of recovery, the number of tuft cells in the crypts was restored to levels comparable to control mice (Fig. 3A). Five days of recovery were required for full restoration of tuft cells to the villi. Enteroendocrine cells were also restored after recovery from BET bromodomain inhibition, but with slower kinetics (Fig. 3B). The enteroendocrine cells were significantly reduced in villi (p < 0.02) and crypts (p < 0.006) after 5 days of BETi treatment. Cell numbers were not fully restored in the crypts until 5 days after the last exposure to BETi. Furthermore, 10 days were required to restore the enteroendocrine cell population in the villi to levels seen in control mice. These results demonstrate that depletion of tuft and enteroendocrine cells after BETi treatment is reversible, and suggests the loss of these cells can be mitigated by modulation of BET bromodomain inhibition.

### Selective loss of progenitor cell markers after BET bromodomain inhibition

**Figure 4:**
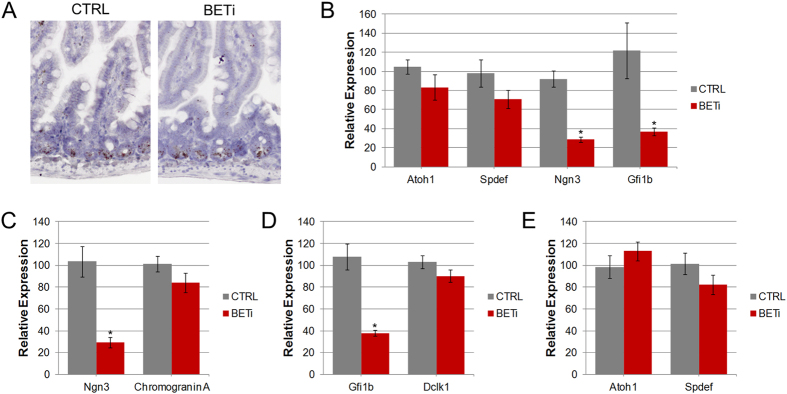
BET bromodomain inhibition selectively affects the transient amplifying cell population. (**A**) Representative images of *in situ* hybridization for Lgr5 in the jejunum after 14 days of treatment with BETi or vehicle control. Original magnification 200X. (**B**) RNA was isolated from purified crypts from the jejunum of mice treated with vehicle control or BETi for 14 days and the expression of Atoh1, Spdef, Ngn3, and Gfi1B were evaluated. The average (±std. dev.) relative expression from three mice is shown. (**C–E**) RNA was isolated from purified crypts from the jejunum of mice treated with vehicle control or BETi for 24 days. The expression of Ngn3 and chromogranin A (**C**), Gfi1b and Dclk1 (**D**), and Atoh1 and Spdef (**E**) were evaluated. Differences in the expression that were statistically significant (p < 0.05) are indicated by an asterisk.

Tuft and enteroendocrine cells are derived from unique populations of transient amplifying (TA) cells that come from common Lgr5-expressing stem cells in the crypts. We therefore evaluated whether BET bromodomain inhibition resulted in changes to the expression of progenitor cell markers in the crypts of the small intestine. Because immunohistochemical analysis of these populations is not possible due to a lack of antibodies or low expression of specific markers, we evaluated these cell populations using the RNA of crypts of mice treated with BETi or vehicle control. *In situ* hybridization was employed to detect the expression of Lgr5 RNA (Fig. 4A). Comparable levels of Lgr5 were detected in crypts of vehicle and BETi treated mice, suggesting the loss of tuft and enteroendocrine cells was not due to the loss of Lgr5-expressing stem cells.

We then examined whether BET bromodomain inhibition affects the TA cells that are dedicated to differentiation into tuft and enteroendocrine cells. Of the pathways required for intestinal cell maturation, the molecular mechanisms by which enteroendocrine cells form are among the best characterized. Enteroendocrine cells are derived from an Atoh1-expressing progenitor cell, from which goblet and Paneth cells are also formed. Atoh1 is not significantly altered (p = 0.23) in mice after BETi treatment (Fig. 4B). Since the ultimate cell fate is established by maturation to TA cells that express either Ngn3, which is required for enteroendocrine cell development, or Spdef, which determines goblet and Paneth lineage, we then evaluated the expression of these TA markers (Fig. 4B). Consistent with the equivalent abundance of goblet and Paneth cells between vehicle and BETi treated mice, no significant change (p = 0.19) in Spdef expression was observed. However, the expression of Ngn3 was decreased by 65% (p < 0.001) in mice treated with BETi, suggesting that BET bromodomain inhibition is affecting the TA cells responsible for enteroendocrine cell differentiation.

To determine whether these diminished levels of Ngn3 were due to reduced Ngn3 expression within the TA cells, or loss of the Ngn3-expressing TA cells, we evaluated the expression of Ngn3 in crypts from mice treated with BETi for 24 hours (Fig. 4C). This short-term BET bromodomain inhibition was sufficient to reduce Ngn3 expression by more than 70% (p < 0.008) relative to control mice. No change in the expression of chromogranin A, a marker of mature enteroendocrine cells, was detected at this time point. Taken together, these results suggest that BET bromodomain inhibition blocks the formation of enteroendocrine cells by down-regulating Ngn3, impairing the function of their dedicated TA cells.

In contrast to enteroendocrine cells, little is known about the regulation of tuft cell differentiation. The only known marker for TA cells that differentiate into tuft cells is Gfi1b, a transcription factor that is expressed in both tuft cell precursor TA cells and fully differentiated tuft cells^22^. However, a functional role for Gfi1b in these cells has not been demonstrated. Evaluation of Gfi1b expression revealed a 70% decrease (p < 0.05) in crypts after 14 days of BET bromodomain inhibition (Fig. 4B). Similarly, Gfi1b expression was decreased by more than 80% after 24 hours of BETi treatment (Fig. 4D). No significant changes in the expression of the tuft cell marker Dclk1 (p = 0.19) or TA cell markers Atoh1 (p = 0.35) and Spdef (p = 0.22) were observed at this early time point (Fig. 4D,E). While the functional significance of altered Gfi1b is not known, these results reveal that BET bromodomain inhibition results in a rapid change in gene expression within the intestinal crypts that selectively effects enteroendocrine and tuft cell differentiation.

### BET bromodomain inhibition sensitizes crypts to gemcitabine-induced apoptosis

**Figure 5:**
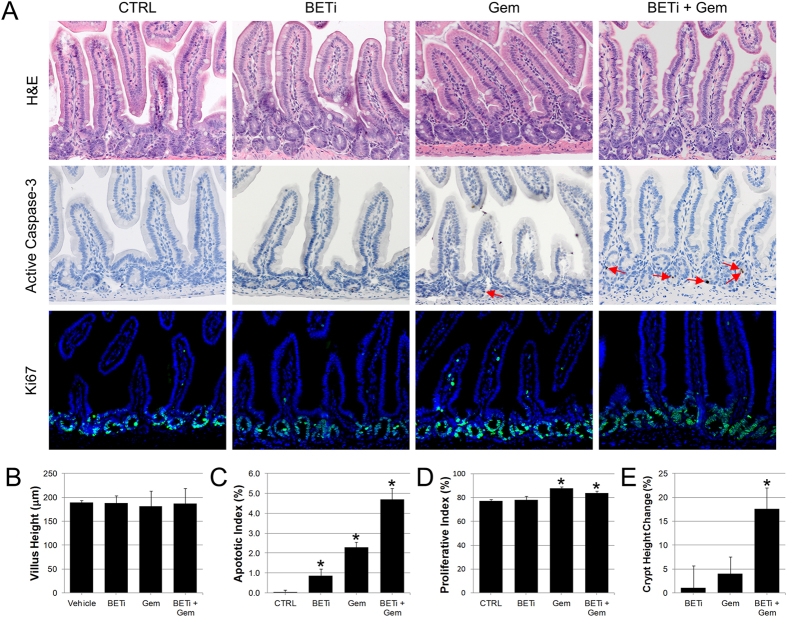
BET bromodomain inhibition increases the sensitivity of the small intestine to chemotoxic stress. (**A**) Representative H&E images, immunohistochemistry for active caspase-3, and immunofluorescence for Ki67 of the small intestine of mice after treatment with vehicle control, BETi, gemcitabine (Gem), or BETi + Gem are shown. (**B**) The villus height (avg. ± std. dev.) measured from 3 control and BETi treated mice are shown. (**C**) The apoptotic index (avg. ± std. dev.) from each group is shown. Significant increases (p < 0.01) in apoptosis relative to control mice are noted by an asterisks. (**D**) The proliferative index (avg. ± std. dev.) from each group is shown. Significant increases (p < 0.01) in proliferation relative to control mice are noted by an asterisks. (**E**) Difference in the height of the crypts from mice treated with BETi, Gem, and BETi + Gem relative to mice treated with vehicle control. The average (±std. dev.) of three mice are shown. Asterisk indicate p < 0.007.

Tuft cells increase in abundance in response to injury in the stomach and intestine, suggesting that they play an important role in homeostasis and regeneration^23^,^24^. Furthermore, loss of tuft cells sensitizes the small intestine to radiation induced injury^25^. Enteroendocrine cells also play a key role in promoting intestinal crypt repair in response to chemotoxic damage^26^. To determine whether BET bromodomain inhibition affects maintenance of the stem cell compartment upon chemotoxic challenge, we evaluated the effects of combined administration of BETi with gemcitabine, a common chemotherapeutic that exhibits GI toxicity^27^,^28^. These experiments employed a 5-days on; 2-days off dosing regimen of BETi, allowing for partial restoration of tuft and enteroendocrine cell populations during the 14 day study period. No gross differences were observed in the histology of the small intestine between mice administered vehicle control, BETi, gemcitabine, or BETi and gemcitabine (Fig. 5A, top row). Average villus length was also unaffected by combined treatment of BETi and gemcitabine, as compared to controls (Fig. 5B). However, active caspase-3 staining revealed that combined administration of BETi and gemcitabine resulted in abundant apoptosis of cells within the crypts (Fig. 5A, middle row). Although BETi and gemcitabine alone induced low levels of active caspase-3 positive cells in the crypts (0.9% and 2.3% respectively), the combination of these agents acted synergistically to induce apoptosis in nearly five percent of crypt cells (p < 0.001) (Fig. 5C). Furthermore, although only small increases in cell proliferation as measured by Ki67 staining was observed in the crypts between treatment groups (Fig. 5A,D), the crypts in the mice treated with BETi and gemcitabine had significantly longer crypts (p < 0.01) than those of mice in the other groups (Fig. 5E). This increase in crypt length is consistent with regeneration in the crypts in response to apoptosis, suggesting that BET bromodomain activity serves a key homeostatic role in maintenance of the stem cell environment in the crypts.

## Discussion

In this study, we report that members of the BET family of proteins are epigenetic regulators of intestinal stem cell differentiation. Treatment with BET bromodomain inhibitors CPI203 or I-BET151 results in selective suppression of tuft and enteroendocrine cells, while leaving enterocyte, goblet, and Paneth cell differentiation unaffected. Suppression of tuft and enteroendocrine cell differentiation is transient and reversible, with these cell types returning after cessation of BET bromodomain inhibition. This block in differentiation results from changes in the transcriptional programs of TA cells that are regulated by BET proteins. Finally, our study shows that inhibition of the BET protein family sensitizes the crypt to chemotoxic damage, as combination therapy with a common chemotherapeutic, gemcitabine, resulted in wide-spread apoptosis in the crypts of the small intestine, indicating a key role for BET bromodomains in homeostatic maintenance of the stem cell compartment.

The recent identification of biomarkers for TA cells specific to each of the absorptive and secretory intestinal cell types has underscored the complex and heterogeneous nature of the intestinal crypt^29^. The migration from crypt base to villus is marked by changes in gene expression that are reflected in the fate of each cell. Instead of a distinct moment of transition from stem cell to mature cell, differentiation is a gradual process, wherein changes in the transcriptional programs in the transiently amplifying cell population occur gradually and cells become more differentiated and specialized as they migrate up from the base of the crypt^30^.

Bolden *et al*. reported that shRNA-mediated silencing of Brd4 results in the loss of all secretory cell lineages in the small intestine^15^. This is in contrast with our study, where BET bromodomain inhibition only results in the loss of tuft and enteroendocrine cells. The key difference between these studies is that Bolden *et al*. reduced the expression of Brd4 while our work specifically targeted the BET bromodomains. These results suggest that BET proteins contribute to intestinal differentiation through both bromodomain-dependent and –independent mechanisms. This is highlighted by the loss of stem cell marker expression in the crypts of mice expressing shRNA targeting Brd4, whereas the abundance of Lgr5-expressing cells is not altered by BET bromodomain inhibition, suggesting that the basic proliferative unit of the stem cell compartment may be regulated by additional functional domains of Brd4.

**Figure 6:**
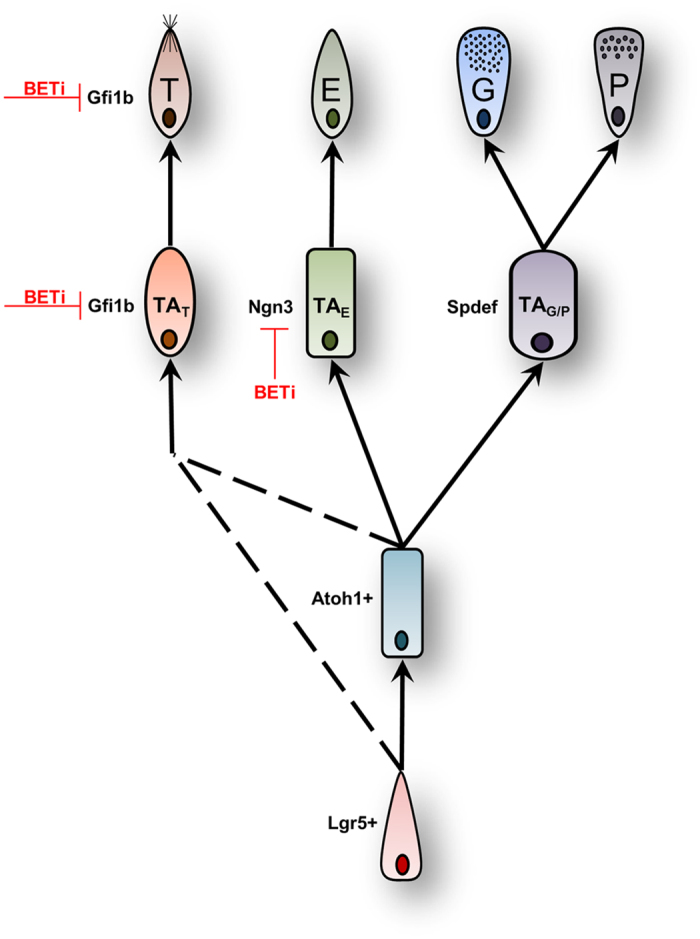
BET bromodomain function in intestinal stem cell differentiation. A schematic representation of the differentiation of Lgr5-expressing stem cells into each of the secretory cell types. Lgr5 + stem cells differentiate into Atoh1 + cells, a precursor for enteroendocrine (E), goblet (G), and Paneth (P) cells. Atoh1 + cells form goblet and Paneth cells though a pathway involving Spdef-expressing TA cells (TAG/P). Enteroendocrine cells are derived from Atoh1 + cells through an Ngn3-dependent intermediate (TAE). The activity of BET bromodomains is required for the formation of TAE cells, or the proper expression of Ngn3 within this cell population. There are conflicting studies that describe Lgr5 + cells forming tuft cells (T) through pathways that are dependent and independent of an Atoh1 + intermediate. The uncertain lineage of tuft cells is represented by dashed lines. Gfi1B is expressed in both TA cells destined for tuft cell formation (TAT) as well as fully differentiated Dclk1-expressing tuft cells. BET bromodomain activity is likely required for the differentiation of TAT cell into mature tuft cell.

While bromodomain-independent roles of Brd4 play a key role in the maintenance of the crypt stem cells, BET bromodomains play a highly selective role in the regulation of the TA cell population (Fig. 6). Atoh1 expression defines one of the earliest TA cell populations, and these cells give rise to either Spdef-expressing or Ngn3 TA cells. However, only the expression of Ngn3 is decreased after BET bromodomain inhibition. Given the key role Ngn3 plays in the differentiation of cells to the enteroendocrine lineage, it is likely that rapid decrease in Ngn3 expression within the TA cells after inhibition of BET bromodomains diminishes their ability to differentiate into mature enteroendocrine cells^11^.

Tuft cells are a relatively new cell type under investigation within the intestine, and little is known about the genetic determinants required for their formation. While other secretory cells of the small intestine are dependent on the Atoh1 lineage, there are conflicting reports on the requirement of Atoh1-expressing cells for the development of tuft cells^12^,^31^. Regardless, our study demonstrates that BET bromodomain inhibition does not alter Atoh1 expression. Our results demonstrating that BET bromodomain inhibition blocks tuft cell formation is the first functional example of a transcriptional pathway regulating tuft cell differentiation. The transcriptional repressor Gfi1b is the only marker that has been attributed to TA population that results in the formation of tuft cells; however Gfi1b expression is also retained in fully differentiated tuft cells. The biological significance of Gfi1b within the TA population or in mature tuft cells has not been elucidated. However, a role for Gfi1b in the differentiation of hematopoietic cells has been established. The deletion of Gfi1b in mice as well as its suppression in cultured cells inhibits the terminal differentiation of hematopoietic progenitor cells into erythroid cells and megakaryocytes^32^,^33^,^34^,^35^. Germline deletion of Gfi1b invariably results in embryonic lethality, in large part to its role in erythropoiesis and megakaryopoiesis^33^,^35^. Given the important role of Gfi1b in hematopoiesis, and our observation that after BET bromodomain inhibition Gfi1b expression is decreased prior to loss of tuft cells, underscores the need for further functional characterization of Gfi1b in tuft cell development.

While BET bromodomain inhibition employing CPI203 and I-BET151 results in dramatic reductions in tuft and enteroendocrine cells, these effects were not reported in studies using a different BET bromodomain inhibitor, JQ1^15^. However, the authors of this study only reported the examination of the Paneth cell population after JQ1 treatment. Interestingly, the addition of low concentrations of JQ1 to *in vitro* organoid assays prevented intestinal organoid formation, suggesting that BET bromodomain inhibition may have a larger effect on the biology of crypts *in vitro* than *in vivo*^15^.

The results of our study reveal a unique role for BET bromodomains in the formation of tuft and enteroendocrine cells. The observation that inhibition of BET proteins sensitizes the crypt to chemotoxic challenge indicates the importance of this family in maintenance and protection of the stem cell environment. These effects may extend beyond chemotoxic agents and have important implications in regenerative responses to other forms of injury. Administration of the BET bromodomain inhibitor JQ1 dramatically increased the damage in response to colitis induced by dextran sodium sulfate (DSS)^36^. Similar to these results, depletion of Dclk1 + tuft cells dramatically exasperated the effects of DSS colitis. Although the effects of JQ1 in tuft cell formation have not been reported, it is likely the increased damage observed in this study were at least partially due to the loss of tuft cells^25^. The increasing entry of pharmacological inhibitors of BET proteins into the clinic as targeted cancer therapies emphasizes the need for a greater understanding of the function of these proteins in normal homeostasis and repair. Future studies examining BET bromodomain inhibition in Lgr5, Gfi1b and Ngn3 reporter mice, will help to elucidate the role of BET bromodomains in regulation of the intestinal stem cell compartment.

## Material and Methods

### Mouse samples

Experiments were performed in accordance with the Office of Laboratory Animal Welfare and approved by the Institutional Animal Care and Use Committee at the Massachusetts General Hospital. BET bromodomain inhibitor CPI203 (BETi) (10 mg/kg) or vehicle control were administered by intraperitoneal injection to 6–7 week old B6:129 mice (Jackson Laboratory, Bar Harbor, Maine) twice daily for 1, 5, 7, or 14 days. I-BET151 (30 mg/kg) was similarly administered once a day for 14 days. Gemcitabine (40 mg/kg) was administered to mice twice a week by intraperitoneal injection. Proliferating cells were labeled with bromodeoxyuridine (BrdU) (100 mg/kg) given by intraperitoneal injection 2 hours prior to euthanasia.

For *in vivo* intestinal permeability, mice were food fasted for 6 hours prior to gastric gavage administration of phosphate buffer saline (PBS, pH 7.2) containing fluorescein isothiocyanate (FITC)-dextran (4 kDa) (Sigma, Saint Lois, MO) at a dose of 300 mg/kg body weight. Three hours after gavage, blood was collected by cardiac puncture and the serum was used to assess the level of FITC-dextran as an indicator of intestinal permeability as previously described^37^.

### Intestinal crypt and villus isolation

Intestinal crypts and villi were isolated as previously reported^38^. Briefly, the jejunum from 6–8 week old B6:129 mice were opened longitudinally and washed with ice-cold PBS to remove the luminal contents. Villi were removed by scraping the luminal surface into ice-cold PBS. Villi were collected from the supernatant by centrifugation at 300 × g for 5 min. Crypt fractions were prepared by rinsing the intestines with ice-cold PBS, and cutting them into 2–4 mm pieces. The fragments were washed in 20 ml ice-cold PBS with gentle pipetting until the supernatant was almost clear (5–10 washes). Fragments were incubated in 30 ml of ice-cold PBS containing 2 mM EDTA for 30 min at 4 °C. Crypts were released by pipetting with ice-cold PBS. Washing in ice-cold PBS was repeated until most of the crypts were released, as determined by microscopic analysis. Crypt suspensions were passed through a 70 μm cell strainer and centrifuged at 300 × g for 5 min. RNA and whole cell lysates from Villi and crypts was prepared using an RNeasy Plus mini-kit (Qiagen, Germantown, MD) and RIPA buffer, respectively. For intestinal alkaline phosphatase assays, whole cell lysates from villi were used to determine protein levels and intestinal alkaline phosphatase (IAP) enzyme activity as previously described^37^.

### Western blot analysis

Whole cell lysates were resolved on Novex 3–8% Tris-Acetate gels (Life Technologies, EA0378BOX), transferred to nitrocellulose, and probed with antibodies specific to Brd2 (Bethyl Laboratories, A302-583A), Brd3 (Bethyl Laboratories, A302-368A), Brd4 (Bethyl Laboratories, A301-985A50), Villin (Santa Cruz Biotech, sc-7672) and PCNA (Santa Cruz Biotech, sc-9857AC). Following incubation with appropriate horseradish-peroxidase conjugated secondary antibodies, membranes were developed using Pierce ECL Plus Substrate (Life Technologies, Grand Island, NY).

### Gene expression analysis

Total RNA isolated from villi and crypts was analyzed by qPCR using TaqMan RNA-to-Ct 1-Step Kit (Applied Biosystems) with the LightCycler® 96 System (Roche). qPCR analyses were performed with TaqMan assays (Applied Biosystems) specific to PHF15 (Mm01294665), Dclk1 (Mm00444950_m1), chromogrnain A (Mm00514341_m1), and Ngn3 (Mm00437606_s1), Gfi1b (Mm00492318_m1), Spdef (Mm00600221_m1), and Atoh1 (Mm00476035_s1). Reactions were normalized to parallel TaqMan assays analyzing the expression of β2-microglubulin (Mm00437762_m1).

### Histology, Immunohistochemistry, and Immunofluorescence

Specimens were harvested and fixed overnight in 10% formalin/phosphate-buffered saline and embedded in paraffin blocks. The proliferative capacity of the small intestine and apoptosis were determined by immunohistochemistry for BrdU (Abcam, ab6326), Ki67 (Abcam, ab15580) and active caspase-3 (Cell Signaling Technologies, 9579S). The cellular composition of the intestine was determined using antibodies for Dclk1 (Abcam, ab37994), chromogranin A (Abcam, ab15160), Muc2 (Santa Cruz Biotech, sc-15334), lysozyme (Dako, A0099), and synaptophysin (Abcam ab32127). Biotinylated secondary antibodies were applied at 1:1000 dilutions and visualized by using diaminobenzidine peroxidase (DAB) substrate (Invitrogen, Carlsbad, CA). Slides were counterstained with hematoxylin. For immunofluorescent staining, secondary antibodies were applied at 1:500 dilution and nuclei were stained with 4′,6-diamidino-2-phenylindole (DAPI).

### *In situ* hybridization

*In situ* hybridization for Lgr5 was performed using RNAscope 2.0 high Definition – Brown assay (Advanced Cell Diagnostics, Hayward, CA) according to manufacturer’s instructions. Slides from at least 3 mice treated with vehicle control or CPI-203 for 14 days were stained with probes specific to Lgr5 (#312171). Slides were also stained with positive and negative control probes (#313911 and #310043, respectively).

### Quantification of cells

From each of 3 control and BETi treated mice, one hundred villi and underlying crypts were selected for analysis. The number of positive stained cells in crypts and villi were counted and the height of each villus was measured using ImageJ (v1.48, National Institute of Health, USA). The average number of positive cells per villus was normalized to the average villus height. The use of villus height and crypt number to normalize cell counts was validated by counting the total number of cells from 5 villi and 10 crypts in each of 3 control and BETi treated mice. No significant difference in the density of cells was observed in villi (control = 39.2 ( ± 1.9) cells/100 μM of villi length; BETi = 41.2 ( ± 0.6) cells/100 μM of villi length (p = 0.15) or crypts (control = 20.1 ( ± 0.5) cells/crypt; BETi = 19.6 ( ± 1.0) cell/crypt (p = 0.47)).

Total and caspase-3 positive cells from at least 30 cross sections of well formed crypts were counted from 3 mice, for a total of 100 crypts for each treatment group. Apoptotic index was calculated by dividing capsase-3 positive cells by the total number of cells per crypt and then multiplying by 100. For crypt height determination, photographs (200X magnification) were taken of least 33 longitudinally cut crypts from each of 3 mice. Crypt height was measured by importing these images into Image J software and drawing a vertical line at the horizontal center of the crypt from the top of the crypt to the base. All statistical analyses were performed using two-tailed Student’s t-tests. Statistically significant changes were defined as those with p ≤ 0.05.

## Additional Information

**How to cite this article**: Nakagawa, A. *et al*. Selective and reversible suppression of intestinal stem cell differentiation by pharmacological inhibition of BET bromodomains. *Sci. Rep*. **6**, 20390; doi: 10.1038/srep20390 (2016).
